# Nitrous Oxide/Whippits-Induced Thoracic Spinal Cord Myelopathy and Cognitive Decline With Normal Serum Vitamin B₁₂

**DOI:** 10.7759/cureus.24581

**Published:** 2022-04-29

**Authors:** Bahadar S Srichawla

**Affiliations:** 1 Neurology, University of Massachusetts (UMass) Chan Medical School, Worcester, USA

**Keywords:** substance use disorder, drug addiction, public health, psychiatry, methylmalonic acid, vitamin b12, nitrous oxide abuse, nitrous oxide myelopathy, whippits, neurology

## Abstract

Abuse of nitrous oxide leads to irreversible neurologic deficits. Nitrous oxide is commonly abused through the inhalation of whipped cream canisters. These whipped cream chargers, also known as "whippits," are widely available in the United States and their sale is unregulated. There is an increasing prevalence of whippet abuse, and many are unaware of the catastrophic effects. The mechanism of injury is mediated through severe depletion of vitamin B₁₂. Here, we report a case of nitrous oxide abuse leading to thoracic cord myelopathy. The patient has been inhaling approximately 80-100 nitrous oxide canisters daily for three months total. A magnetic resonance imaging (MRI) of the spinal cord illustrates the lesion within the thoracic cord. Further diagnostic workup with serum vitamin B₁₂ levels was normal. However, elevated levels of methylmalonic acid (MMA) were seen. Despite supplementation of vitamin B₁₂, the patient’s neurologic deficits persisted, and he was referred to a rehabilitation center. The abuse of whippets is a serious public health threat and warrants greater regulation of their sale. MMA and holotranscobalamin (holoTC) are improved biomarkers for diagnosing vitamin B₁₂ deficiency.

## Introduction

Nitrous oxide has an increasing prevalence as a recreational drug. Nitrous oxide is stored in steel cartridges and is used in the culinary industry in whipped cream dispensers. These whipped cream dispensers are being misused as recreational drugs through the inhalation of nitrous oxide gas found in the canister. Thus, these whipped cream chargers got the iconic term "whippits" amongst users of the drug and are a significant public health threat [1].

Inhalation of nitrous oxide leads to severe vitamin B₁₂ deficiency, causing profound effects on multiple organ systems, but most notably in the central nervous system (CNS). Some effects on the CNS include subacute combined degeneration, myelopathy, mood disorders, and dementia. Diagnosis of whippits-induced neurologic deficits involves checking serum, vitamin B₁₂, and neuroimaging to correlate symptoms to a lesion [2]. Sometimes, vitamin B₁₂ may be normal and the diagnosis can be missed. Checking holotranscobalamin (holoTC) and methylmalonic acid (MMA) levels improve diagnostic markers. Both of these biomarkers have improved sensitivity and specificity in detecting vitamin B₁₂ deficiency, especially when used together in comparison to vitamin B₁₂ alone.

Myelopathy is defined as a lesion to the spinal cord and can have varying presentations depending on the neuroanatomical tracts that are affected. The dorsal or posterior column of the spinal cord allows for vibratory sensation and proprioception. The spinothalamic tracts provide pain and temperature sensory input, and the lateral corticospinal tract allows for motor function. Spinal cord compression, vertebral fractures, disk protrusion, and nutritional deficiencies are common causes of myelopathy. Vitamin B₁₂ deficiency often causes both sensory and motor deficits, known as subacute combined degeneration. Subacute combined degeneration occurs due to lesions to both the corticospinal and dorsal column tracts. Neuroimaging plays an important role in diagnostics and therapeutic interventions depending on the underlying cause, and permanent neurologic dysfunction is not uncommon [3]. Vitamin B₁₂ deficiency has also been implicated in both acute cognitive decline, confusion, and dementia. Often times, supplementation with vitamin B₁₂ does not improve symptoms.

Here, we report a case of nitrous oxide-mediated myelopathy secondary to inhalation of whipped cream canisters with a normal level of serum vitamin B₁₂. The patient's symptoms were primarily motor with significant hyperreflexia, lower extremity clonus, and rigidity. Magnetic resonance imaging (MRI) demonstrated primarily central cord involvement, correlating with symptomatology. This case aims to articulate the catastrophic long-term neurologic deficits of whippit abuse and to address genuine public health threats related to it. We also advocate for refined diagnostic criteria using holotranscobalamin and methylmalonic acid in the diagnosis of vitamin B₁₂ deficiencies and their reasoning.

## Case presentation

A 44-year-old male with no previous medical history presented to the emergency room with a chief complaint of sub-acute onset upper and lower extremity rigidity and memory loss. Symptoms first presented four weeks ago and have gradually worsened. The bilateral upper extremities were more affected than the lower extremities. The rigidity worsened, and the patient began using a cane to walk approximately one week prior to the presentation. The patient was questioned about his history of drug usage and endorsed the inhalation of whipped cream canisters (whippits). The patient had been inhaling approximately 80-100 canisters of nitrous oxide daily on average for the past three months. The patient denied all other recreational drug usage. There was no recent history of trauma. The patient followed an omnivore diet, including meat, milk, and cheese. A comprehensive neurologic exam showed diffuse 3+ hyperreflexia of the bilateral upper and lower extremities with a down-ward Babinski reflex. A five-to-six-beat ankle clonus was seen bilaterally. The patient had bowel and bladder continents. Other findings included clasp-knife rigidity of the upper extremities and generalized rigidity of the lower extremities with an active range of motion. Sensation testing, including vibratory, temperature, pin-prick, and proprioception, was intact. Romberg’s test was negative. The patient had an ataxic gait. A mini-mental status exam (MMSE) was completed, showing mild-cognitive decline with a score of 22. Specific deficits on the MMSE were in object recall and calculation. The patient tested negative for severe acute respiratory syndrome coronavirus 2 (SARS-CoV-2) ribonucleic acid (RNA) via a polymerase chain reaction (PCR) test. A comprehensive urinary toxicology screen was negative.

**Figure 1:**
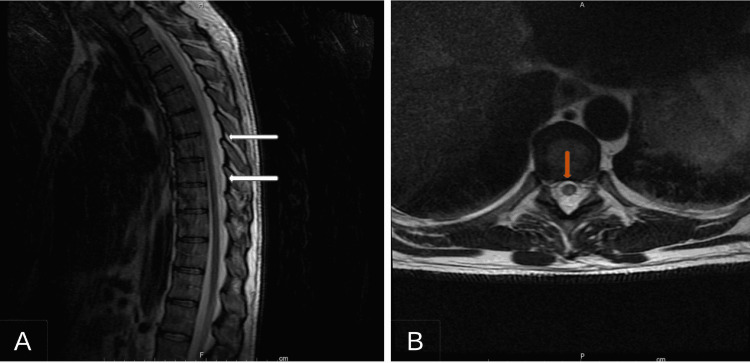
MR imaging of the thoracic spine T2/FLAIR sagittal view (A) Sagittal view of the thoracic spinal cord and (B) axial view of the thoracic spinal cord.

A complete blood count (CBC), comprehensive metabolic profile (CMP), serum vitamin B₁₂, and folate were obtained with values within normal ranges. The Vitamin B₁₂ level was 304 pg/mL (normal 180-904 pg/mL). With and without contrast, magnetic resonance imaging (MRI) of the brain, cervical, thoracic, and lumbar spine was obtained. An MRI of the thoracic spinal cord revealed significant and diffuse increased T2 signal abnormalities throughout the central portion of the spinal cord. T2-weighted-fluid-attenuated inversion recovery (T2/FLAIR) is shown in Figure 1. An MRI of the brain showed no abnormalities.

Further diagnostic workup included homocysteine, MMA, vitamin B6, vitamin E (alpha and B-gamma), intrinsic factor (IF) blocking antibody, Lyme panel, and copper levels. CSF was not obtained. Significant results included elevated homocysteine of 97.5 µmol/L and MMA of 2980 nmol/L. Holotranscobalamin (holoTC) was not available at this institution. The patient was diagnosed with nitrous oxide-induced vitamin B₁₂ deficiency and was started on high-dose intramuscular (IM) injections of vitamin B₁₂ at 1000 mcg daily for ten days total. Further workup was completed to rule out additional etiologies of vitamin B₁₂ deficiency, including TSH, free T4, anti-TPO antibodies, and hepatitis panel, which were negative or within normal limits. Daily neurologic examinations were completed. On day 10, the patient’s focal neurologic deficits, including hyperreflexia, rigidity, and clonus, persisted. A repeat of the MMSE was significant for a score of 23. Given the poor outcome, the patient was referred to a rehabilitation facility and was maintained on daily oral vitamin B₁₂ of 100 mcg henceforth.

## Discussion

Nitrous oxide is a colorless, odorless, and non-irritating gas. Nitrous oxide (N_2_O) is a gaseous agent that is used in whipped cream canisters. Recreational inhalation of these whipped cream canisters, better known as whippits by those who abuse them, has a growing prevalence. In the United Kingdom, whippits are the second most commonly used recreational drug, second only to cannabis. Whippits are a widely available item sold in retail stores and online. There are currently limited regulations on its sale, which have led to increased recreational abuse [4]. However, the question remains: what effect does N2O have on its users, encouraging them to abuse it? Humphry Davy was the first to use nitrous oxide as an anesthetic agent, and in 1844, Horace Wells used it as an inhaled anesthetic for dental extraction. Clinical trials have shown that N_2_O has a positive effect in treating refractory depression. N_2_O has hallucinogenic properties and induces euphoria [5]. The effects of N_^2^_O only last a few minutes, and minimal hangover symptoms are seen. Although the euphoric effects of N_2_O last only a few minutes, long-term abuse of N_2_O leads to irreversible neurologic deficits.

Nitrous oxide affects multiple organ systems, however, most notable is its neurotoxic effects. Upon inhalation of nitrous oxide, it immediately crosses the alveoli and diffuses into the bloodstream. The neurologic deficits seen secondary to N_2_O are hypothesized to be secondary to vitamin B₁₂ deficiency. Some of these neurologic deficits include myelopathy, subacute combined deficiency, delirium, altered mental status, and depression. Myelopathy has variable clinical symptoms based on the location of the lesion along the spinal cord. Common symptoms include numbness/tingling, pain, and descending weakness. In our case, the patient presented with primarily motor symptoms, including rigidity and hyperreflexia, with minimal sensory involvement. This is an atypical presentation of vitamin B₁₂ deficiency, as commonly, sensory involvement is also seen. MR imaging in our case (Figure 1) showed significant central cord involvement, which is consistent with the neurologic exam findings. Subacute combined degeneration is notable for both lateral and dorsal column lesions. Similarly, other cases of nitrous oxide abuse have primarily shown motor symptoms [6]. Further follow-up on the patient's clinical course may reveal the development of sensory symptoms. The use of whippits in this case was for three months, a shorter duration than what has been reported in other cases and of a higher volume of approximately 80-100 canisters per day. Maheshwari and Athiraman also recorded a high level of whippits abuse at approximately 600 canisters per day [6]. Other cases of whippits/nitrous oxide abuse have shown spinal cord myelopathy with both sensory and motor symptoms [7]. Prognosis in patients with myelopathy is difficult to predict; however, significant focal neurologic deficits on the initial exam, as well as symptoms being present for a longer duration, are poor clinical markers. In our patient, T2 hyperintensities were seen throughout much of the thoracic cord, and symptoms had persisted for a month prior to presentation, indicating a poor prognosis [8].

Vitamin B₁₂ also known as cobalamin, is one of eight B vitamins that play a key role in multiple cellular processes. One mechanism of toxicity is through the oxidation of vitamin B₁₂ from its active form (+1) to an inactive form (+2), reducing concentrations of methyl-cobalamin and causing decreased enzymatic activity of methionine synthetase (MTR). MTR plays an important role in generating methionine from homocysteine, a key regulator of many biosynthetic and regenerator pathways [9]. Another proposed mechanism of neurotoxicity includes vitamin B12-mediated dysregulation of myelinotoxic and myelinotrophic cytokines. An increased ratio of myelinotoxic cytokines may lead to the loss of myelin and subsequent neuronal dysregulation [10].

Currently, there are no screening guidelines for vitamin B₁₂ (cobalamin). However, they can be considered in patients with inflammatory bowel disease, small bowel resection, older adults, and strict vegans. Serum vitamin B₁₂ has a sensitivity between 95% and 97% and MMA at >95%. Although serum vitamin B₁₂ has a high sensitivity, various medical conditions can falsely elevate B₁₂ levels. Some of these diseases include alcohol abuse and cirrhosis [11]. Vitamin B₁₂ has three distinct carrier proteins: trans-cobalamin, IF, and haptocorrin. Liver dysfunction causes decreased clearance of these carrier proteins and leads to falsely elevated vitamin B₁₂ levels. Haptocorrin can be lower in pregnant patients, which can confound total vitamin B₁₂ levels in this patient population. Thus, total vitamin B₁₂ is an inconsistent biomarker for cobalamin. Therefore, other lab markers should be used when there is a high suspicion of vitamin B₁₂ including macrocytosis and MMA.

Holotranscobalamin is an emerging diagnostic marker used to more readily detect vitamin B₁₂ deficiency. Approximately 25% of cobalamin binds to holoTC and it is also known as active vitamin B₁₂ [12]. Fluctuations in these binding proteins do not affect holoTC as they do with cobalamin levels. Utilization of trans-cobalamin and methylmalonic acid together in detection for the detection of vitamin B₁₂ has a sensitivity of >99%. However, despite the improved diagnostic criteria, holoTC is not widely available at numerous institutions [13]. Common agents of drug-induced vitamin B₁₂ include metformin, proton pump inhibitors, and histamine-2 receptor antagonists such as famotidine. Drugs of abuse associated with cobalamin deficiency include N2O, amphetamines, and alcohol abuse [14]. Myelopathy is a clinical diagnosis made based on the pertinent history, symptoms, and findings of neuroimaging.

**Figure 2:**
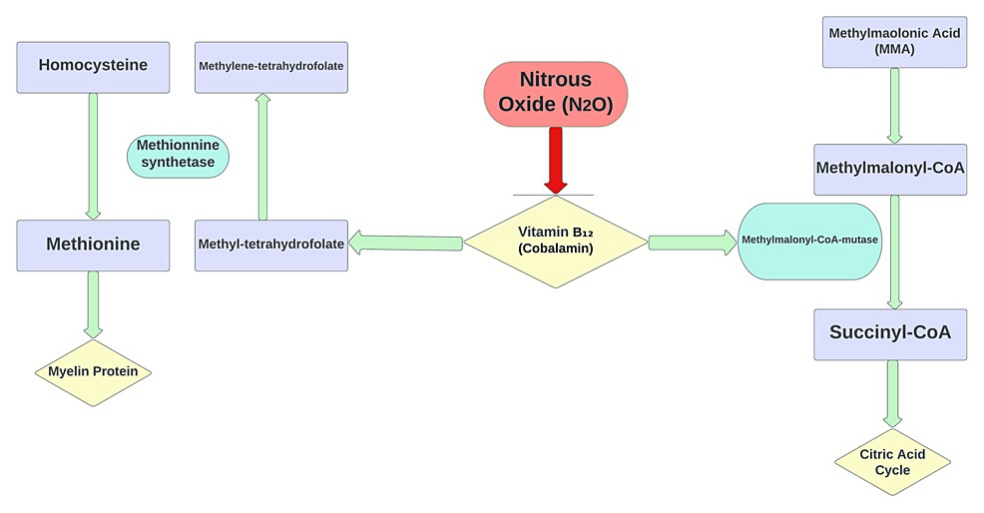
Nitrous oxide-mediated inhibition of vitamin B₁₂

Structural abnormalities such as vertebral fractures, disc herniation, spinal stenosis, and spinal cord transection are common causes of myelopathy. Non-structural etiologies of myelopathy include auto-immune, infectious, vascular, metabolic/nutritional, and drug-induced [15]. N_2_O is a drug of abuse that causes cobalamin deficiency as previously described and can lead to myelopathy. The mechanism of myelopathy occurs through demyelination secondary to cobalamin deficiency; however, the exact mechanism is not well understood. One study points toward the inhibition of homocysteine methyltransferase as a potential cause [16]. Other studies have hypothesized the accumulation of propionyl-CoA and fatty acids as a cause of axonal injury and demyelination. Newer studies have shown that increased levels of tumor necrosis factor-alpha (TNF-α) and interleukin-6 (IL-6), as well as lower levels of epidermal growth factor (EGF), are present, which sheds new light on the mechanism of demyelination [17]. Vitamin B₁₂ deficiency is also implicated in the pathogenesis of neurocognitive deficits both in the short and long term. Increased MMA and homocysteine levels increase the risk of developing Alzheimer’s disease (AD), and decreased serum vitamin B₁₂ levels have been associated with acute neurocognitive impairment. In our case, the patient presented with the sub-acute onset of mild cognitive decline with a score of 22 on the MMSE, which had only improved to a score of 23 with IM cobalamin supplementation, which is within the margin of error. It would be necessary to further follow the patient's clinical trajectory with further rehabilitation to check for cognitive improvement. Cobalamin supplementation does not seem to improve cognitive impairment secondary to vitamin B₁₂ deficiency [18]. Figure 2 illustrates the key biochemical pathways involving vitamin B₁₂.

Vitamin B₁₂ deficiency is more commonly seen in the elderly. Approximately 6% of adults over 60 years old in the United States and the United Kingdom have low cobalamin levels identified through high MMA and homocysteine levels. The most common cause of vitamin B₁₂ deficiency in the elderly is malabsorption. In developing countries, malnutrition is the most prevalent etiology of cobalamin deficiency. The most common routes of vitamin B₁₂ supplementation include oral, intramuscular, and sublingual. Management of cobalamin deficiency is typically done with 1000 mcg of oral cobalamin. In patients with intrinsic factor deficiency or those with neurologic deficits, intramuscular vitamin B₁₂ 1000-2000 mcg can be administered weekly [19]. In our case, the patient was treated with IM vitamin B₁₂ 1000 mcg daily for ten days and then transitioned to oral vitamin B₁₂ on discharge, with no improvement in symptoms. Whippits abuse leads to irreversible neurologic dysfunction, as reported in this case and various others in the scientific literature. Further legislative efforts to regulate the in-person and online sale of whipped cream canisters (whippits) on a national and global level. For example, in New York, state legislation (S.2819-A) prohibits the sale of whipped cream canisters to people under the age of 21.

## Conclusions

We report a case of nitrous oxide abuse with approximately 80-100 whippits per day for three months. The patient presented with severe stiffness and hyperreflexia of the extremities with minimal sensory involvement. MR imaging of the thoracic spine demonstrated full cord involvement, however, most pronounced within the central cord. Further follow-up with serial neurologic exams is required to monitor the evolution of symptoms, including the development of sensory deficits, which are commonly seen in vitamin B₁₂ deficiency. The utilization of MMA and holoTC are more sensitive and specific biomarkers in detecting vitamin B₁₂ deficiency. Although the exact mechanism of injury is not known, N_2_O causes cobalamin deficiency and disrupts the biosynthesis of various cofactors involved in the regulation of cellular proteins. Nitrous oxide abuse is a significant ongoing public health threat that leads to potentially irreversible neurologic dysfunction. Greater regulatory efforts are needed to control the sale and distribution of whippits. Further studies are needed on public health initiatives that spread awareness of the long-term effects of nitrous oxide abuse.
